# Clinical Profile of HIV/AIDS-infected Patients Admitted to a New Specialist Unit in Dhaka, Bangladesh—A Low-prevalence Country for HIV

**DOI:** 10.3329/jhpn.v29i1.7562

**Published:** 2011-02

**Authors:** Nashaba Matin, Lubaba Shahrin, Mohammed Moshtaq Pervez, Sayera Banu, Dilruba Ahmed, Mahmuda Khatun, Mark Pietroni

**Affiliations:** ICDDR, B, GPO Box 128, Dhaka 1000, Bangladesh

**Keywords:** Acquired immunodeficiency syndrome, HIV, Tuberculosis, Retrospective studies, Bangladesh

## Abstract

This paper describes the clinical features of a series of patients admitted to the specialist HIV/AIDS unit (Jagori) of the Dhaka Hospital, ICDDR, B (International Centre for Diarrhoeal Disease Research, Bangladesh) during May 2008–February 2010. Data were collected from a review of documents and electronic case-records and collation of laboratory results with respect to CD4 counts. One hundred and nine patients were admitted during this period. Their mean age was 33.4 years, and 62% were male. On admission, the mean CD4 count±standard deviation (SD) was 244±245 (range 2-1, 549). The death rate was 12%. The patients were classified as World Health Organization clinical stage 1: 23%, stage 2: 30%, stage 3: 23%, and stage 4: 24% during the admission. The commonest diagnosis recorded was tuberculosis (TB) (23%), which was also the commonest cause of death (38%). Even for those clinicians with limited experience of managing AIDS cases, the commonest problem encountered in this patient group was TB, reflecting the continued high burden of TB on health services in Bangladesh. Additional challenges to managing TB/HIV co-infection include atypical presentations in HIV-infected persons and the complex drug interaction with antiretroviral therapy.

## INTRODUCTION

Although the prevalence of HIV remains very low (<0.1%) in the general population and low (<1%) in most at-risk populations (MARPs) (1, 2), by the end of 2009, the Ministry of Health and Family Welfare (MoHFW) in Bangladesh had confirmed 1,745 HIV cases, 619 of which had developed acquired immunodeficiency syndrome (AIDS), and 204 had died (3). The national AIDS/STD Programme of the MoHFW estimated that 7,500 people in Bangladesh are living with HIV (3).

In the developed world, the management of HIV/AIDS has been transformed by the widespread availability of antiretrovirals and management of opportunistic infections. In Bangladesh, a limited range of (first line) antiretrovirals has been available since 2003 (3). A specialist inpatient unit (Jagori) for the management and treatment of HIV-positive individuals was launched in May 2008 in the Dhaka Hospital of ICDDR, B. Managing HIV/AIDS is a relatively new field in Bangladesh, and little information exists regarding the clinical presentation of AIDS in the country. This paper reviews the clinical profile of patients admitted to the Jagori Unit during May 2008–February 2010.

## MATERIALS AND METHODS

This is a retrospective review of all the admissions to the Jagori Unit of the Dhaka Hospital of ICDDR, B during May 2008–February 2010.

In Bangladesh, a number of peer non-governmental organizations (NGOs) provide care and support to people living with HIV (PLHIV). The vast majority of the admissions to the ward are referred from these NGOs and so are previously known to be HIV-positive. Admission data for the May 2008–February 2010 period were gathered from an admission notebook kept on the ward, and the paper notes were reviewed for clinical and microbiological data. Since February 2009, the Dhaka Hospital moved to an electronic patient-record system (EPR), and admission data were gathered from this computerized system. All microbiological and virological data were extracted either from the EPR or from the chart review. Diagnosis data and World Health Organization (WHO) clinical staging data were extracted from the clinical notes.

*Mycobacterium tuberculosis*-associated infection (TB) was diagnosed using the following criteria: pulmonary TB was diagnosed by appropriate clinical symptoms and/or a chest radiograph compatible with TB and a positive sputum smear for acid fast bacilli or a positive sputum culture report. Disseminated TB was classified as clinical features of TB and involvement of at least two organs, with bacteriological or histological evidence of TB. TB meningitis was diagnosed by bacteriological evidence on cerebrospinal fluid examination or after exclusion of other causes of meningitis and/or with radiological evidence (computerized tomography brain imaging—CT) of TB meningitis and clinical improvement after the commencement of anti-tuberculosis therapy. Malignancies were confirmed histologically. Oesophageal candidiasis was diagnosed by endoscopic examination or clinical evidence of oral candidiasis with swallowing difficulties which improved on antifungal therapy. Cytomegalovirus (CMV) retinitis was diagnosed on fundoscopic examination; CMV colitis was diagnosed by suggestive clinical symptoms and colonoscopic evidence of ulceration which resolved on Ganciclovir therapy. The diagnosis of disseminated fungal disease was made on histological examination of biopsy samples. The diagnosis of sepsis was made for patients who presented with the clinical features of septicaemia, which was either confirmed bacteriologically and/or responded to antibacterial treatment.

All antiretroviral prescribing was carried out by the patient's affiliated NGO; so, limited data were available on regime switches and reasons. Data were extracted for drug regime on admission, new diagnoses of HIV during admission, and the reasons for carrying out an HIV test.

## RESULTS

One hundred and nine patients were admitted to the ward during May 2008–February 2010. Their age ranged from 1.5 years to 65 years (mean 33.4 years and median 35 years). Sixty-eight (62%) patients were male, and 41 (38%) were female. The adultpatients were aged 18-65 years (mean 35.3 years, median 36 years, and mode 40 years).

Data regarding the history of previous risks of HIV acquisition were collected from the medical notes. Heterosexual transmission was recorded in 87 (80%) of the cases. Forty (37%) patients reported a history of external migration (living abroad for employment purposes) in the past. This group of patients comprised exclusively males. Thirty-two (29%) had HIV-positive spouses, of whom most (97%) were exclusively female. Ten (9%) patients were admitted previously, who reported buying sex from sex workers. Five (5%) patients gave a history of previous commercial sex work. After heterosexual transmission, a history of injecting drug-use was recorded in 11 (10%) cases and possible vertical transmission (mother also found to be positive) in 6 (5.5%) cases. Two (1.8%) cases had a history of bisexual contact or being MSM (men who have sex with men). In two (1.8%) cases, no risk-factor data were available, and one (0.9%) was believed to be transfusion-associated. Seven patients (6.4%) were children (defined as age below 18 years); their age ranged from 1.5 years to nine years (Table 2). The mode of transmission for six of the children was believed to be vertical; one child had received multiple blood transfusions from an HIV-infected donor and had an HIV-negative mother.

During the admission/upon discharge, the patients were classified according to clinical stages of the WHO (4) as follows: clinical stage 1: 25 (23%) patients; clinical stage 2: 33 (30%) patients; clinical stage 3: 25 (23%) patients; and clinical stage 4: 26 (24%) patients.

CD4 count data were available for 102 (94%) of the patients (Table 1). CD4 counts were measured upon admission. The mean CD4 count was 244±245 [standard deviation (SD)] (range 2-1, 549 and median count 170). Of the 109 admitted patients, 13 (12%) died in hospital. Five (38%) deaths occurred due to TB (3 cases of pulmonary TB, one case of disseminated TB, one case of TB meningitis). Four (31%) deaths were attributed to overwhelming sepsis, one (7.7%) case was due to CMV disease, and one (7.7%) case due to malignancy. Two (15%) cases had no attributable cause. Of the 13 deaths, eight had a CD4 count of <50 cells/µL.

**Table 1.** CD4 count results (specimens were taken on admission)

CD4 cells/µL	No. of patients (n=102)		CD4 counts for in-hospital deaths (n=13)	
	No.	%	No.	%
<50	24	24	8	62
51-200	32	31	2	15
201-350	19	19	1	8
>351	27	26	2	15

Among all the patients, the most common diagnosis recorded was TB with 25 cases (23%) (Table 2). The second commonest diagnosis was pulmonary infection, with 12 (11%) cases recorded. Some patients were recorded as having more than one diagnosis. Eight women were admitted with pregnancy-related conditions, i.e. shortly after caesarean section for postnatal care.

**Table 2.** Opportunistic infections and/or HIV-related conditions diagnosed in both adult and paediatric patients (patients may have had more than 1 diagnosis)

Diagnosis	Cases	
	No.	%
Tuberculosis—all	25	23
Pulmonary	16	
Disseminated	5	
TB meningitis	2	
Lymph node	1	
Joint	1	
Pulmonary infection	12	11
Candidiasis—all	11	10
Oral	3	
Oesophageal	5	
Vulvovaginal	3	
Pregnancy-related admission	8	7.3
Skin—all	8	7.3
Fungal	4	
Drug rash	3	
Infestation (scabies)	1	
Abscess/cellulitis	6	5.5
CMV disease—all	4	3.7
CMV retinitis	3	
CMV colitis	1	
Sepsis	5	4.6
Herpes Zoster	3	2.8
Malignancy—all	3	2.8
Kaposi sarcoma	1	
Poorly-differentiated carcinoma of tongue	1	
Infantile fibrosarcoma	1	
Peripheral neuropathy	3	2.8
Disseminated histoplasmosis	2	1.8
Cryptosporidiosis	1	0.9
Shigellosis	1	0.9
Bacterial meningitis	1	0.9
Recurrent otitis media	1	0.9

CMV=Cytomegalovirus; HIV=Human immuno-deficiency virus; TB=Tuberculosis

A range of diagnoses not related with AIDS was recorded (Table 3). The commonest diagnosis recorded was diabetes mellitus (8.2%), followed by hypertension (3.7%).

**Table 3.** Diagnoses not related with AIDS (pat-ients may have had more than one diagnosis)

Diagnosis	Cases	
	No.	%
Diabetes mellitus	9	8.2
Hypertension	4	3.7
Urinary tract infection/renal colic	3	2.8
Physical assault, e.g.street-fight	3	2.8
Haemoglobinopathy	2	1.8
Psychological distress	2	1.8
Surgical, e.g. appendicitis	2	1.8
Ischaemic heart disease	1	0.9
Cerebral stroke	1	0.9
Hypothyroidism	1	0.9

In 100 (92%) cases, the patients were known to be HIV-positive before admission to hospital. Nine patients were newly diagnosed to be HIV-positive. They were inpatients in our own hospital (n=6) or another healthcare facility (n=3), with another medical condition. The conditions that led to carrying out an HIV test are outlined here. One case was diagnosed with Kaposi's sarcoma, and in three cases, there was a diagnosis of TB in conjunction with a history of injecting drug-use in one case, an associated history of professional sex work in one case, and a history of buying sex from sex worker in one case. In three other cases, TB was diagnosed, and no additional indication for testing was recorded. In two patients, the test was carried out due to a long history of loss of weight and intractable diarrhoea for which no pathogen had been identified. Forty-five (41%) patients were on antiretroviral therapy at the time of admission. Twenty-six (24%) patients were started on antiretrovirals while admitted on our unit. All but two of the 71 patients (97%) were on a nucleoside reverse trans-criptase inhibitor (NNRTI)-based regimen, along with a nucleoside backbone. Of those who started antiretrovirals while in hospitals, CD4 counts were available for 24 patients. Two of the 24 patients had a CD4 count of <200 cells/µL. One patient had a CD4 count of 210 cells/µL and a diagnosis of a pulmonary infection. The second patient had a CD4 count of 575 cells/µL and was diagnosed with pulmonary TB.

## DISCUSSION

Most (94%) patients admitted to the Jagori Unit were adults. The small number of paediatric admissions hopefully reflects a small number of children infected with HIV in Bangladesh but little data exist on the actual prevalence of paediatric HIV in the country.

The largest group of patients in our cohort reported heterosexual transmission. In 37% of the admissions, a previous history of external migration was recorded.

Recent data from our own voluntary counselling and testing (VCT) centre within the Jagori Unit of ICDDR, B demonstrated that, of the male clients who were HIV-positive, over 70% were returning migrant workers from overseas. The vast majority travel to the Middle East or other countries in South Asia for employment. Mercer *et al*. reported that extramarital sex was 2-3 times higher in spouses who live apart (5). The proportions of men who reported sex with a female sex worker (51%) or with another male (5.4%) while living away were also higher. Subtyping of HIV suggests that these infections were acquired from abroad. The true prevalence of HIV infection in this client group as a whole is unknown, and effective intervention measures to prevent HIV infection among returning migrants and their spouses require further study.

In our cohort, the second largest group of patients had no other recorded risk for HIV transmission other than an HIV-positive spouse (29%). This group comprised exclusively females. However, one cannot ascertain who was infected first—the patient or the spouse. The proportion of patients with a previous history of injecting drug-use (IDU) was 10%. The prevalence data in Bangladesh revealed a concentrated epidemic of HIV among IDUs, with prevalence rates up to 7% (1). Lower rates of admissions of IDUs may reflect referral practices for them or differing healthcare-seeking behaviour among this client group. Bisexuality or MSM practices were recorded on only two oc-casions. Societal attitudes within Bangladesh may discourage patients from admitting this to healthcare practitioners. Only one patient was thought to be infected by a blood transfusion. Unsafe blood transfusion is a matter of concern in Bangladesh where professional blood donors still donate blood to earn extra income. They are more likely to be infected with bloodborne viruses than voluntary donors, with one report quoting a hepatitis B surface antigen positivity rate of 29% vs 4% in voluntary donors (6). National blood-screening recommendations are in place in Bangladesh but monitoring of blood banks to ensure adherence to the recommendations needs strengthening.

With respect to CD4 count data, the largest number of patients was in the group with CD4 counts of 51-200 cells/µL (31%). The second largest group had CD4 counts of above 350 cells/µL (26%). Although those with a CD4 count of <50 cells/µL represented 24% of the cohort, 62% of deaths were in this group of patients, indicating high morbidity and mortality associated with low CD4 counts, which has been previously reported (7).

TB was the most frequently-recorded diagnosis and the commonest attributable cause of death in our series. Worldwide, it is estimated that 8% of new TB cases are attributable to HIV infection, and data from 2006 showed that 200,000 persons globally died from HIV-TB co-infection (8). Despite the advent of highly-active antiretroviral therapy, TB is commonly diagnosed in HIV patients in other case series within Asia. Three other case series reflect a much higher TB diagnosis rate than we encountered in our patient-group. A series from Thailand reported 42% prevalence of TB (9) while two case series from India reported TB infection rates of 52% and 55% (10, 11).

Bangladesh is a high-burden country (387 per 100,000 population) for TB (12), and so one would expect that we would have seen rates of TB prevalence closer to those quoted in other studies from the region. The lower prevalence in this group of admitted patients may possibly reflect a lower prevalence in our cohort of patients; however, this seems unlikely when one considers the socioeconomic context of Bangladesh, the high prevalence of TB in Bangladesh, and also what is already known about the epidemiology of HIV-TB co-infection. We conjecture that it may, in part, reflect the steep learning curve for doctors who are trying to diagnose TB, which can present atypically in HIV-infected patients (13-15).

In Bangladesh, chest x-ray and sputum smear microscopy for acid fast bacilli (AFB) are widely available but culture facilities are limited to a handful of centres (including our centre). TB in Bangladesh is commonly diagnosed by suggestive clinical symptoms and signs coupled with a suggestive chest x-ray and sputum sample that is smear microscopy-positive for AFB. HIV-positive patients with active TB may still have a normal chest x-ray and may have sputum specimens that are smear-negative for AFB; however, the sputum may subsequently be culture-positive. It is important that clinicians retain a high index of suspicion for TB, especially in patients with suggestive symptoms, despite initial investigations being normal.

Overall, the prevalence of TB in this patient-group we describe here only represents those who were ill enough to warrant hospital admission and will, thus, represent the tip of the iceberg in terms of TB-HIV co-infection in Bangladesh. As the cohort of HIV-infected patients grows in Bangladesh and the experience of managing these cases increases, we expect to see a proportionate increase in the number of diagnosed TB cases in this patient-group.

Antiretroviral treatment in Bangladesh is primarily based on non-nucleoside reverse transcriptase inhibitor regimes with limited availability of protease inhibitors (PI). Drugs for the treatment of TB are usually supplied by the National TB Programme free of charge to the healthcare facility but rifabutin is not available through this programme if required for a patient on a PI-based regime. In common with many other developing countries, the limited choice of ARVs and the well-described interactions between TB drugs and ARVs through the cytochrome P450 system in the liver (16) present additional challenges to managing TB-HIV co-infection in this setting.

Oral or oesophageal candidiasis was only reported in eight patients. The mean CD4 count in this group was 244, which may explain the lower numbers seen, as this tends to present more often with CD4 counts of <200 cells/µL (17).

Certain common opportunistic infections are not described in this case series. The lack of pneumocystis pneumonia diagnoses may be due to a number of reasons. Co-trimoxazole prophylaxis against pneumocystis infection is effective (18) and widely available and prescribed to patients newly diagnosed with HIV presenting with a CD4 count of <200 cells/µL. In addition, diagnostic facilities to diagnose pneumocystis are at present limited in Bangladesh; so, it is possibly being underdiagnosed. In the case of fungal infections, two cases of disseminated histoplasmosis were diagnosed on histological examination of a lymph node biopsy and a skin biopsy respectively. There are no cases of cryptococcal disease noted. Despite widespread reports of penicilliosis in South-East Asia (19) and India (20), none was identified in our series. There is, at present, limited availability of fungal stains and/or culture and cryptococcal antigen tests in Bangladesh, which can limit the ability to diagnose this group of conditions.

As the experience of managing HIV patients in Bangladesh increases, it is hoped that the demand for and availability of these tests will increase. Cerebral toxoplasmosis is often diagnosed radiologically in the context of clinical history and signs. No cases were diagnosed in the above cohort. No data on the prevalence of toxoplasma infection in Bangladesh are available, although case series from neighbouring India quoted a figure of 31% in healthy volunteers and a prevalence of 68% in HIV-infected patients (21). It is not clear why we have not observed any cases of cerebral toxoplasmosis, although the widespread use of co-trimoxazole prophylaxis may explain this to some degree. Among the diagnoses not associated with HIV, the incidence of diabetes mellitus in our case series was 8.2%, which reflects urban estimates of type 2 diabetes in Bangladesh (22).

Most (92%) patients are known to be HIV-positive upon referral to this unit; however, nine (8%) patients were diagnosed while in the hospitals. Testing of HIV antibody is not routinely carried out in all hospitals in Bangladesh, and many hospital facilities do not have the laboratory capability to carry out HIV testing. Late diagnosis of HIV infection has been associated with increased mortality and morbidity due to HIV (23). Many undiagnosed HIV patients with advanced disease may present to healthcare facilities on multiple occasions, and the diagnosis will be missed (24). As knowledge and understanding about HIV becomes more widely known, it is hoped that inpatient HIV counselling and testing will be more widely available in healthcare settings in Bangladesh.

### Conclusions

Bangladesh is still at the beginning of an evolving epidemic of HIV-associated disease. This case series serves to describe the spectrum of HIV-associated disease and opportunistic infections in Bangladesh where currently there is a paucity of data in this field. Healthcare providers in Bangladesh are rising to the challenge of managing this emerging disease in the country but are still at the beginning of the learning curve in how to manage HIV. The lack of diagnostic facilities in many healthcare facilities in Bangladesh poses an additional challenge to those wishing to manage opportunistic infections in this patient-group. While technical capacity is increased to manage this condition nationwide, strong referral pathways between district health facilities and specialist centres must be created and nurtured. Des-pite the challenge of dealing with a relatively new disease in Bangladesh, the commonest problem we encountered was still from *M. tuberculosis*-associated infection. It is important to avoid developing vertical HIV programmes that do not interact well with the pre-existing healthcare infrastructure that is in place to control TB in Bangladesh. A good link with the national TB-control programmes must be maintained to ensure uninterrupted supply of treatment materials for TB and also to ensure minimal interactions with antiretroviral therapy for HIV-TB co-infected patients. This link will be an important tool in the fight against TB in this already-high burden nation.
